# High-power sub-terahertz source with a record frequency stability at up to 1 Hz

**DOI:** 10.1038/s41598-018-22772-1

**Published:** 2018-03-12

**Authors:** Andrey Fokin, Mikhail Glyavin, German Golubiatnikov, Lev Lubyako, Mikhail Morozkin, Boris Movschevich, Alexander Tsvetkov, Gregory Denisov

**Affiliations:** Institute of Applied Physics of the Russian Academy of Sciences (IAP RAS), Nizhny Novgorod, 603950 Russia

## Abstract

Many state-of-the-art fundamental and industrial projects need the use of terahertz radiation with high power and small linewidth. Gyrotrons as radiation sources provide the desired level of power in the sub-THz and THz frequency range, but have substantial free-running frequency fluctuations of the order of 10^−4^. Here, we demonstrate that the precise frequency stability of a high-power sub-THz gyrotron can be achieved by a phase-lock loop in the anode voltage control. The relative width of the frequency spectrum and the frequency stability obtained for a 0.263 THz/100 W gyrotron are 4 × 10^−12^ and 10^−10^, respectively, and these parameters are better than those demonstrated so far with high-power sources by almost three orders of magnitude. This approach confirms its potential for ultra-high precision spectroscopy, the development of sources with large-scale radiating apertures, and other new projects.

## Introduction

In recent years, considerable progress has been achieved in the development of high-power radiation sources – gyrotrons operating in the sub-terahertz and terahertz frequency range. Interest in high-frequency sources with a medium (up to kW) output power is stimulated by the rapid growth of the number of applications. Gyro-devices are promising for application in radar studies, in particle temperature measurements using the collective Thomson scattering of gyrotron radiation, for synchronization of a large number of high-power THz sources, and as sources for spectroscopy and diagnostics of various media, for example, dynamic nuclear polarization-enhanced nuclear magnetic resonance (DNP-NMR) spectrometry. Microwave driven DNP experiments are now recognized as the most effective and versatile methods of enhancing signals in solid-state and solution NMR and imaging^1^. DNP improves the sensitivity of NMR spectra by about a factor of 100, thus reducing the acquisition time in multidimensional NMR^2,3^. This enhancement permits the study of larger molecules, response dynamics, or high-throughput screening. The power level desired from a THz source in DNP is highly dependent on several factors including the polarization mechanism, sample temperature and volume, polarizing agent, and coupling efficiency, and is now recognized to be at a level of one hundred watts.

Currently, the most widespread devices in the THz frequency range^4^ are backward-wave oscillators (BWOs) that provide an output power of a few milliwatts in the CW regime at the highest admissible frequencies with half-power bandwidth of spectral distribution less than 100 mHz (a relative resolution of about 10^−13^)^5^. The devices based on multiplication of a signal from solid-state sources^6^ with relative linewidths of about 10^−12^ produce subterahertz and terahertz radiation at a power level from hundreds of microwatts to a milliwatt. Quantum cascade lasers already provide a power of hundreds of milliwatts at frequencies down to a few THz^7^ with a possible spectrum width of radiation at the sub-Hz level^8,9^. At the same time, the power of radiation that can be produced by vacuum electron devices based on the stimulated Bremsstrahlung radiation (free-electron lasers (FELs)^10,11^ and gyrotrons^12,13^) can be higher by many orders of magnitude. However, FELs utilize ultrarelativistic electron beams and, hence, typically require large-size particle accelerators for their operation. Unlike FELs, cyclotron resonance masers can operate with electron beams having significantly lower energies, usually 15–70 keV. Cyclotron Resonance Masers (CRMs) represent a wide class of high-frequency electron devices based on stimulated (induced) radiation of electromagnetic waves^14^. Gyrotrons^15^, being the most efficient version of CRMs, are based on interaction of electrons tracing out helical trajectories in a homogeneous magnetostatic field with fast electromagnetic waves under the cyclotron resonance condition; they are much more compact and cheaper than FELs and can be employed in many laboratories.

## Results

### Frequency stabilization of a gyrotron

In the theory of gyrotron operation^16^, the equations of motion of electrons can be reduced to the equations describing a non-isochronous oscillator under the action of a resonance force:

1 $$\frac{da}{d\varsigma }-ia({\rm{\Delta }}-1+{|a|}^{2})=i{a}^{\ast (s-1)}f(\varsigma )F.$$

Here, *a* is the orbital momentum of electrons, normalized to its initial value, the function $f(\varsigma )$ describes the axial structure of the cavity field, $\varsigma =(\pi \,{\beta }_{\perp 0}^{2}/{\beta }_{z0})(z/\lambda )$ is the normalized axial coordinate, where λ is the wavelength,${\beta }_{\perp 0,}{\beta }_{z0}$ are, respectively, the orbital and axial electron velocities normalized to the speed of light. The electron motion depends on the normalized parameters, including the dimensionless amplitude of a high-frequency field, $F=\frac{A}{{B}_{0}}\frac{{\beta }_{\perp 0}^{s-4}}{{\gamma }_{0}}\frac{{s}^{s-1}}{{s}^{s-1}s!}{J}_{m-s}({k}_{\perp }{R}_{b})$ and the cyclotron resonance mismatch ${\rm{\Delta }}=\frac{2}{{\beta }_{\perp 0}^{2}}\frac{\omega -s{{\rm{\Omega }}}_{H}}{{\omega }_{s}}$, where *B*_0_ is the external magnetic field, *γ*_0_ is the initial Lorentz factor of the electrons, *R*_*b*_ is the radius of the electron beam in the cavity, *s* is the cyclotron harmonic number, ${{\rm{\Omega }}}_{H}$ is the electron cyclotron frequency, and ${\omega }_{s}$ is the cold cavity frequency. The field amplitude and frequency in the stationary regime are defined by the balance equations for imaginary and real parts of the complex susceptibility of an electron beam,

2 $$\begin{array}{c}{I}_{0}\hat{\chi }^{\prime\prime} =1\\ {I}_{0}\hat{\chi }^{\prime} =\frac{{\omega }_{s}-\omega }{{\omega }_{s}/2Q},\end{array}$$

where *I*_0_ is the normalized current parameter, and the susceptibility of an electron beam is determined by the motion of electrons in the presence of a high-frequency field as

3 $$\hat{\chi }=\frac{2}{|{F}^{2}|}\frac{1}{2\pi }{\int }_{0}^{2\pi }({\int }_{0}^{\mu }{a}^{\ast s}{e}^{i{\theta }_{0}}{f}^{\ast }(\varsigma ){F}^{\ast }d\varsigma )d{\theta }_{0}.$$

It follows from Eqs (1)–(3) that the output power and frequency depend on a number of parameters, including the magnetic field, the accelerating voltage, the beam current, and the electron pitch factor ($g={\beta }_{\perp }/{\beta }_{\parallel }$); thus, the stability of the output parameters in a gyrotron is defined by deviations of the power supplies. The typical relative linewidth of free-running gyrotrons is about $2\cdot {10}^{-6}$ for high-power devices^17^ and about $7\cdot {10}^{-9}$ for low-power setups^18^. Based on the full theory of the technical noise effects on gyrotron operation^19^, the methods of control and stabilization can be divided into several groups:

- Active methods utilize automated control of the electron beam parameters such as
  - Magnetic field tuning, which varies the cyclotron frequency of the electrons. This method has a limited speed of response by the maximum sweep rate of the cryomagnet or the skin effect in the gyrotron body^20^;
  - Accelerating voltage variation as a conventional way of stabilization by variation of the electron energy, thus changing the electron cyclotron frequency because of the relativistic effect along with the changes in the interaction length and normalized current. The drawback of this method is a need of complex and expensive power supplies with high voltage and current, capable of fast variation of parameters. The known experiments on frequency stabilization of gyrotrons that utilize phase lock control of the accelerating voltage show that the relative width of the frequency spectrum ${\rm{\Delta }}f/f$ and the frequency fluctuations $\delta f/f$ can be lower than $1\cdot {10}^{-9}$ and $3\cdot {10}^{-10}$, respectively^21^;
  - Modulating anode voltage variation permits one to change the electron pitch factor without changing the electron energy: $g={1/(\frac{2e{U}_{0}{B}_{0}^{2}}{m{E}_{c}^{2}{\alpha }^{3}}-1)}^{1/2}$. Here, *E*_*c*_ is the electric field on the cathode, which is varied by the anode voltage, *U*_0_ is the accelerating voltage and α is the magnetic compression ratio^22^. Variation of the electron pitch factor perturbs the electron motion, changing the active and reactive components of susceptibility and, therefore, changes the power^23^ and operating frequency of the gyrotron^24^;
- Passive methods based on lock by an external signal^25^ or the impact of the reflected signal on the gyrotron operation^26^.

In our experiment, we used the anode voltage variation as a way of frequency control and stabilization, since a low anode current reduces the power supply requirements and a small capacitance of the modulating anode relative to other electrodes increases the speed and performance of the control system.

### Experimental setup

**Figure 1:**
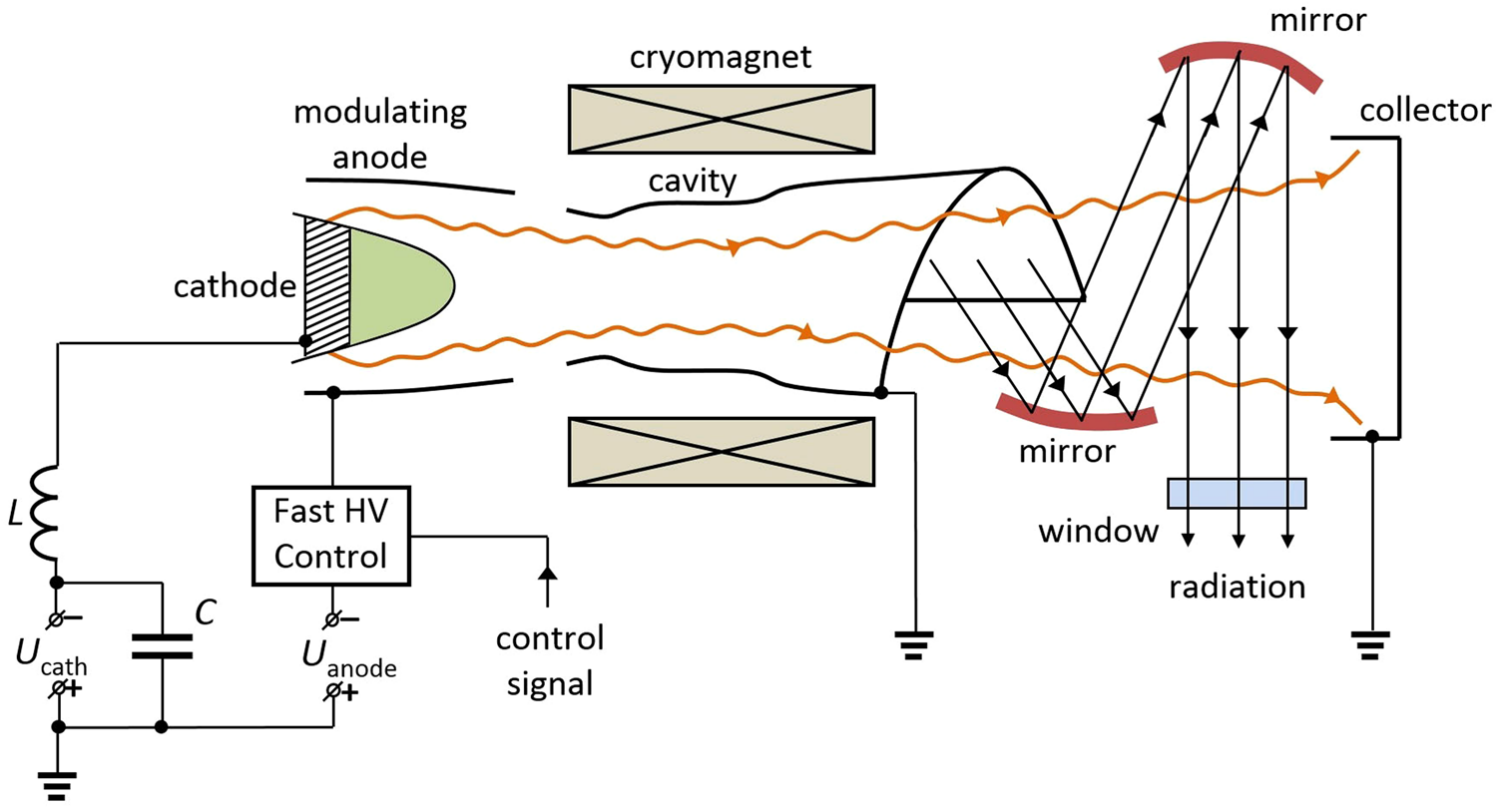
Scheme of the gyrotron and power supply connections.

The experiment on frequency stabilization was carried out using a continuous-wave (CW) gyrotron for spectroscopy and various media diagnostics with an operating frequency of 263 GHz and an output power of up to 1 kW for a 15 kV/0.4 A regime^27,28^ with the electron beam formed by a triode-type magnetron injection gun. The gyrotron was designed for operation with JASTEC JMTD-10T100, a liquid helium-free cryomagnet, at the TE_5,3_ mode of a cylindrical cavity. The internal mode converter transforms the operating mode into a Gaussian beam. A simplified scheme of the gyrotron and power supplies connection is presented in Fig. 1.

**Figure 2:**
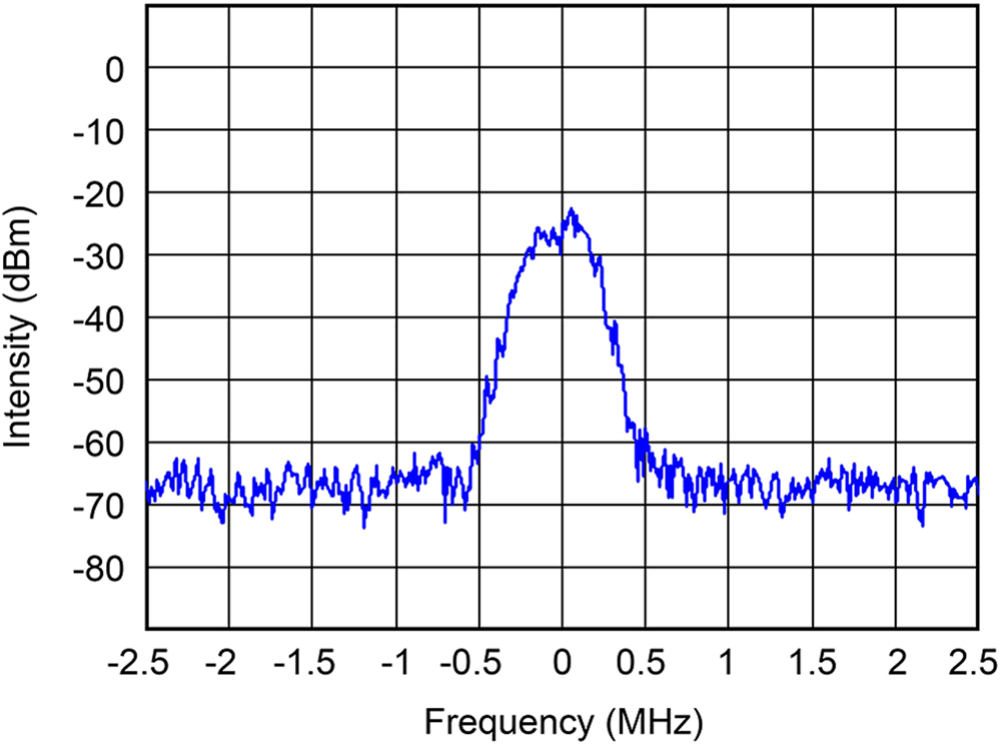
Spectrum of a free-running gyrotron.

The electron beam current chosen for the experiment was 0.2 A and the magnetic field was adjusted so as to set the output power at a level of 100 W as required for spectroscopy. The spectrum width of the output radiation of a free-running gyrotron without stabilization is about 0.5 MHz (see Fig. 2).

For the above-mentioned setup, the main source of the spectrum broadening is the accelerating voltage ripple, which for the CW power supply with a high stability option is voltage fluctuations with ΔU_0_/U_0_ = 0.2% and a frequency band up to 25 MHz. Both the numerical simulations in a self-consistent model and the experiments give a sensitivity of the radiation frequency to the accelerating voltage equal to 33 MHz/kV, in good agreement with the resulting spectrum width of a free running gyrotron, which is defined by the accelerating voltage ripple. The theoretical and experimental sensitivity to the modulation anode voltage is 29 MHz/kV.

In the experiment, we used a phase-locked loop (PLL) against the reference oscillator in order to control the gyrotron modulating anode voltage. A specially designed fast voltage control unit performed voltage modulation in a range of 1 kV with speed better than 1 kV/µs. The voltage drop on the active element of the unit is proportional to the external control signal. Preliminary testing of the control system showed a modulation bandwidth of 150 kHz, defined by the time constant of the modulating anode circuit.

**Figure 3:**
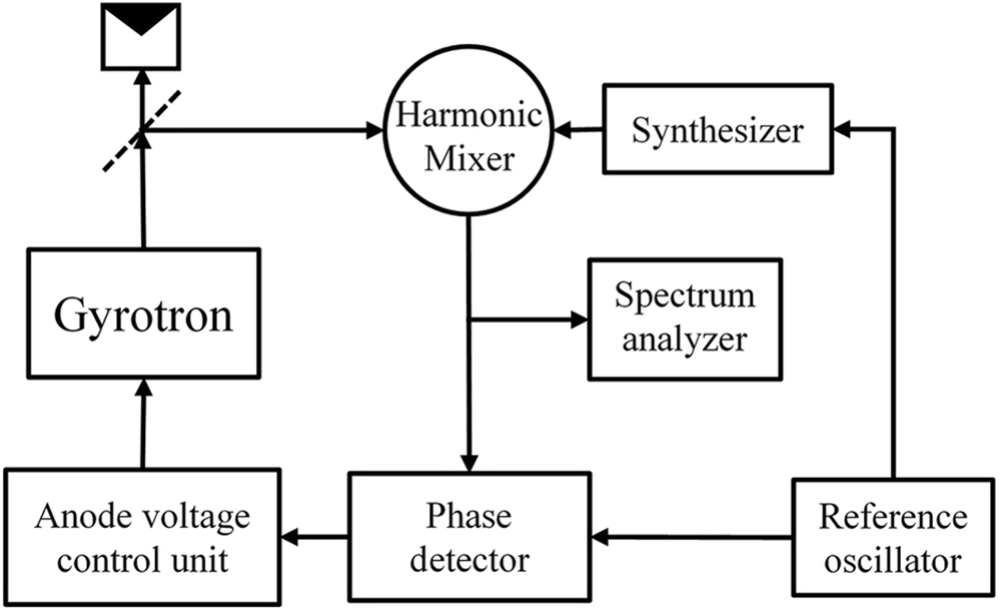
Block diagram of the phase-locked loop units for a sub-THz gyrotron.

A block diagram of the PLL system is presented in Fig. 3. Part of the output radiation of the 263-GHz gyrotron is transmitted to the harmonic mixer, where it is mixed with the 54^th^ harmonic of the signal from a PTS 6400 microwave synthesizer with a frequency of about 4.88 GHz. The resulting signal at an intermediate frequency of 350 MHz is then fed to the phase detector for a frequency-phase comparison with the signal from a quartz clock as the reference oscillator. The error signal from the phase detector is then used as a control signal for the voltage control unit. In addition, the signal at an intermediate frequency is used for spectrum measurements by an R&S FSL spectrum analyzer at 9 kHz-6 GHz.

In order to reduce the high-frequency components of the voltage ripple, which exceed the bandwidth of the control system, a low-pass LC filter was installed in the accelerating voltage circuit. Such a modification precluded the phase lock disruption by high-frequency ripple.

### Results of the experiment

**Figure 4:**
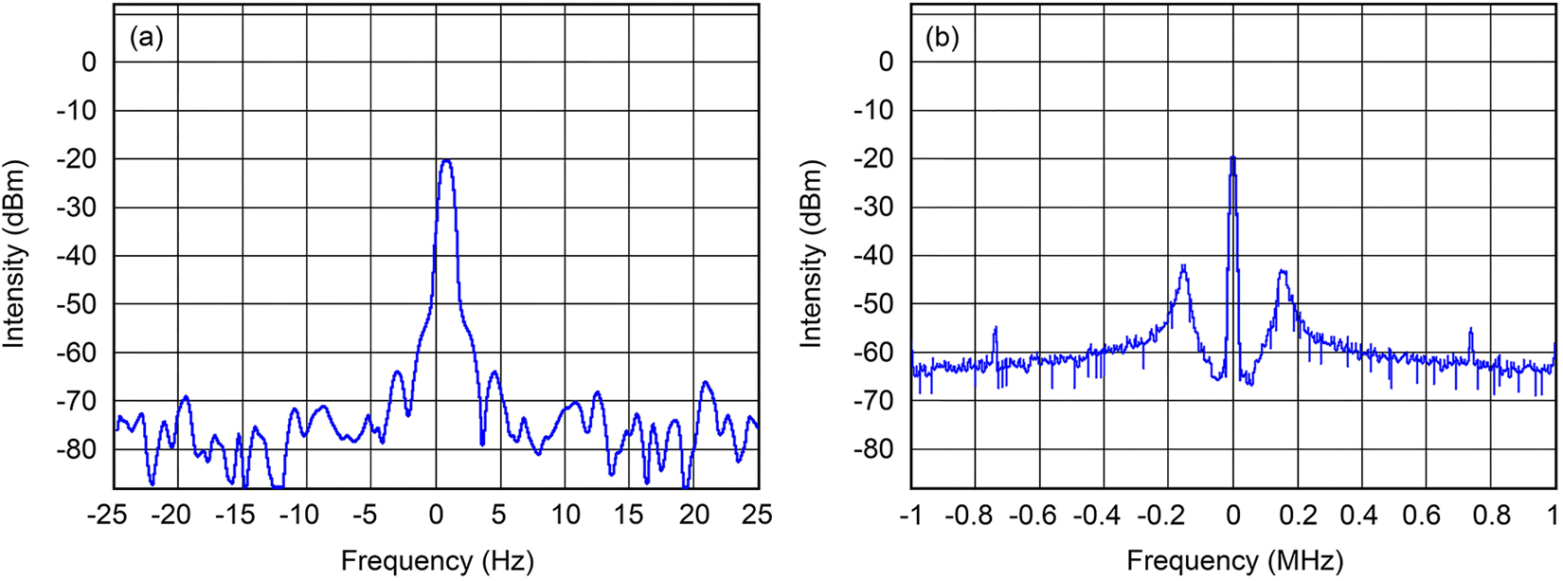
Observed frequency spectrum of the gyrotron with phase-locked loop at an intermediate frequency with spans of 60 Hz (**a**) and 2 MHz (**b**).

Our experimental setup permitted us to control the gyrotron radiation frequency by varying the modulating anode voltage with a modulation frequency of up to 150 kHz. The obtained frequency sensitivity of the gyrotron with a fast voltage control unit was 1 MHz per volt of the control signal in the unit. The total frequency tuning range in the frequency stabilization regime was 4 MHz. After applying the phase-locked loop, the width of the frequency spectrum was decreased from 0.5 MHz for a free-running gyrotron down to 1 Hz for the stabilized gyrotron, measured at the intermediate frequency IF = 350 MHz, which corresponds to ${\rm{\Delta }}f/f=3\,\ast \,{10}^{-12}$ with a measurement time of a few seconds Fig. 4(a). The level of phase noise in the case of a PLL setup for low noise in the 10-kHz offset range can be seen in Fig. 4(b). Single-sideband (SSB) phase noise in the range 10–1000 Hz demonstrates a flat dependence on the offset and does not exceed −60 dBc.

The long-term frequency drift is defined by the stability of the reference oscillator (*δ f/f* ~ 10^−9^ for the quartz clock employed in the experiment) and can be improved by using another reference oscillator with better parameters (for example, a rubidium clock with *δ f/f* ~ 10^−12^)^29^. The output power fluctuations due to a change in the beam pitch factor by the modulating anode voltage were less than the uncertainty of the calorimetric system, and are at a level of ${\rm{\Delta }}P/P=1 \%$.

## Discussion

In the experiment, we used the well-known principle of the phase-locked loop frequency stabilization. Although the concept of the stabilization scheme is similar to that employed in low-power devices such as a BWO, its realization is essentially different. In a BWO, the usual method of frequency control is variation of the accelerating voltage, which for high-power devices like gyrotrons is a complex and rather slow method. Direct control of the cathode potential (as in a BWO^5^) is impeded by the need to vary the parameters of the power supply with both a high voltage and a high current. Variation of the body voltage is slowed down by the relatively high capacitance of the gyrotron cavity relative to other parts; thus, such a method^30^ has a significantly lower bandwidth.

Frequency stabilization by varying the modulating anode voltage combines a low current with a low capacitance, increasing the control system bandwidth while having the same frequency sensitivity as control by accelerating voltage. Furthermore, the dependence of the output power on the modulating anode voltage is weaker than on the accelerating voltage because of the smaller changes in the gyrotron operating parameters. For our experiment, we performed a further optimization of the power supply system of the gyrotron in order to reduce the high-frequency voltage ripple, as well as increase the modulation bandwidth by adjustment of the modulating anode circuit characteristics.

## Conclusions

To conclude, we have achieved phase locking of a 263 GHz gyrotron with an output power of 100 W and a linewidth of 1 Hz, defined mostly by the bandwidth of our spectrum analyzer, for a PLL control bandwidth of 150 kHz. The technique takes advantage of the dependence of the resulting gyrotron frequency on the parameters of the electron beam modulated by an additional low-current anode and has no apparent limiting factors in terms of output power. The demonstrated spectral purity and low phase noise open up new prospects for using THz gyrotrons as new standard sources for spectroscopy. The capability of frequency and phase modulation of the stabilized gyrotron also gives rise to new applications, such as telecommunications and synchronization of a large number of high-power THz gyrotrons.

## Methods

### Gyrotron

The experiments were carried out using the gyrotron for spectroscopy and various media diagnostics produced by GYCOM Ltd. The gyrotron is designed for operation with a JASTEC JMTD-10T100 liquid helium-free cryomagnet. The gyrotron operates at the TE_5,3_ mode of a cylindrical waveguide, which is then transformed to the Gaussian beam by a quasi-optical converter. The electron beam is formed by a triode-type adiabatic magnetron-injection gun. The gyrotron operates with a beam current of 0.02 A to 0.4 A and an accelerating voltage of 15 kV, producing up to 1 kW of radiation with a frequency of 263 GHz for a magnetic field of about 9.6 T. The modulating anode voltage is provided by a separate power supply with a maximum voltage of 5 kV; the gyrotron body is grounded. The power supply system was modified by installation of a low-pass LC filter into an acceleration voltage circuit with L = 160 mH and C = 0.22 µF to reduce the high-frequency voltage ripple; the anode circuit was modified in order to install a voltage control unit. To ensure the maximum modulation bandwidth of the anode voltage control system, the value of the resistors in the modulating anode circuit was reduced to 25 kΩ, limited by ohmic heating, and a non-inductive type of resistors was used.

### Anode voltage control unit

The voltage control unit is designed for fast modulation of voltage with an amplitude of 1 kV and speed better than 1 kV/µs. The active element of the unit is the high voltage pulse tetrode GMI-83V. The tetrode is controlled through an analog opto-isolator using a fast operation amplifier. The high voltage isolation is provided by a 30-cm long optical fiber. Voltage variation in the anode circuit is controlled by an external signal having an amplitude of 0 to 10 V and a linearity of 5% with response time less than 1 µs.

### Data availability

The datasets generated during and during the current study are available from the corresponding author on reasonable request.
